# TMEM16F activation by Ca^2+^ triggers plasma membrane expansion and directs PD-1 trafficking

**DOI:** 10.1038/s41598-018-37056-x

**Published:** 2019-01-24

**Authors:** Christopher Bricogne, Michael Fine, Pedro M. Pereira, Julia Sung, Maha Tijani, Youxue Wang, Ricardo Henriques, Mary K. Collins, Donald W. Hilgemann

**Affiliations:** 1 UCL Cancer Institute, University College London, Gower St, London, UK; 2 University of Texas Southwestern Medical Center, Department of Physiology, Dallas, Texas USA; 3 MRC Laboratory for Molecular Cell Biology, University College London, Gower St, London, UK; 4 National Institute for Biological Standards and Control, Blanche Lane, South Mimms, Herts, UK; 5 Present Address: Okinawa Institute of Science and Technology, Onna-son, Okinawa, Japan

**Keywords:** Cell biology, Membrane trafficking

## Abstract

TMEM16F is a Ca^2+^ -gated ion channel that is required for Ca^2+^ -activated phosphatidylserine exposure on the surface of many eukaryotic cells. TMEM16F is widely expressed and has roles in platelet activation during blood clotting, bone formation and T cell activation. By combining microscopy and patch clamp recording we demonstrate that activation of TMEM16F by Ca^2+^ ionophores in Jurkat T cells triggers large-scale surface membrane expansion in parallel with phospholipid scrambling. With continued ionophore application,TMEM16F-expressing cells then undergo extensive shedding of ectosomes. The T cell co-receptor PD-1 is selectively incorporated into ectosomes. This selectivity depends on its transmembrane sequence. Surprisingly, cells lacking TMEM16F not only fail to expand surface membrane in response to elevated cytoplasmic Ca^2+^, but instead undergo rapid massive endocytosis with PD-1 internalisation. These results establish a new role for TMEM16F as a regulator of Ca^2+^ activated membrane trafficking.

## Introduction

Eukaryotic cells retain phosphatidylserine (PS) on the cytoplasmic face of the plasma membrane^1^. In response to high levels of cytoplasmic Ca^2+^ elevation and during apoptosis, PS is exposed on the cell surface by a process known as phospholipid scrambling^2^. Ca^2+^-activated phospholipid scrambling is mediated by a transmembrane protein, TMEM16F (also known as anoctamin 6)^3,4^, a widely expressed member of the TMEM16 family of ion channels^5^. In platelets, Ca^2+^-activated PS exposure is an important step in blood coagulation, and the main phenotype caused by TMEM16F mutations in humans is a bleeding disorder called Scott Syndrome^3,6^. In contrast, PS exposure during apoptosis, which marks cells for removal by phagocytosis^7^, does not depend on TMEM16F^3^.

There is evidence that TMEM16F itself acts as both a Ca^2+^-activated ion channel and phospholipid “scramblase”. These two functions are activated simultaneously following a large increase in intracellular Ca^2+^ ^4^. Recent work employing single molecules of purified mouse TMEM16F incorporated into membrane microarrays^8^, as well as reconstitution experiments of a fungal TMEM16 homologue in liposomes^9,10^, support the idea that TMEM16F can facilitate bidirectional diffusion of phospholipids. The crystal structure of the fungal TMEM16 reveals a hydrophilic groove that may serve as the channel for both ions and lipids^10^. In addition, a 35 amino acid scrambling domain identified in TMEM16F can confer this function on TMEM16A, a relative of TMEM16F that does not scramble phospholipids^4,11^.

Mice lacking TMEM16F suffer from immune exhaustion leading to a failure to clear viral infections^12^. This is at least in part due to a failure to down-regulate PD-1, which is highly expressed on T cells in TMEM16F-null mice during lymphocytic choriomeningitis virus (LCMV) infection^12^. PD-1 is a negative regulator of the immune system, which can be hijacked by various cancers to evade anti-tumour immune responses; antibodies that block PD-1 function are potent cancer therapeutics^13^. Thus, TMEM16F-dependent regulation of PD-1 expression on the cell surface may be of immunological and clinical significance. The mechanism by which PD-1 expression on T lymphocytes is regulated by TMEM16F has not been explained.

A role for TMEM16F in membrane trafficking has, however, been reported in several other cell types. For example, TMEM16F is involved in microvesicular release from platelets and neutrophils^14^. Mice lacking TMEM16F show deficits in bone development^15^, a process that is linked with microvesicle release from osteoblasts^16^. In macrophages TMEM16F is necessary for phagocytosis stimulated by the ATP receptor P2X_7_^17^ and microglia lacking TMEM16F demonstrate defects in process formation and phagocytosis^18^.

In T lymphocytes, the triggering of the antigen receptor causes a large cytoplasmic Ca^2+^ elevation that can be mimicked by addition of an ionophore such as ionomycin^19^. In this paper we show that cytoplasmic Ca^2+^ elevations induced by ionomycin cause rapid plasma membrane expansion in parallel with PS exposure in Jurkat T cells. The absence of TMEM16F blocks both membrane expansion and PS exposure in response to elevated cytoplasmic Ca^2+^, which surprisingly leads instead to massive endocytosis of the plasma membrane in the cells lacking TMEM16F. We further find that in Jurkat T cells, PD-1 is selectively shed in vesicles after TMEM16F activation, while it is selectively internalised in the absence of TMEM16F. This behaviour of PD-1 depends on its transmembrane region sequence, and other membrane proteins did not show such behaviour. These results provide a potential explanation for the TMEM16F regulation of PD-1 cell surface expression described previously^12^.

## Results

### Ca^2+^ influx triggers PS exposure and plasma membrane expansion

**Figure 1:**
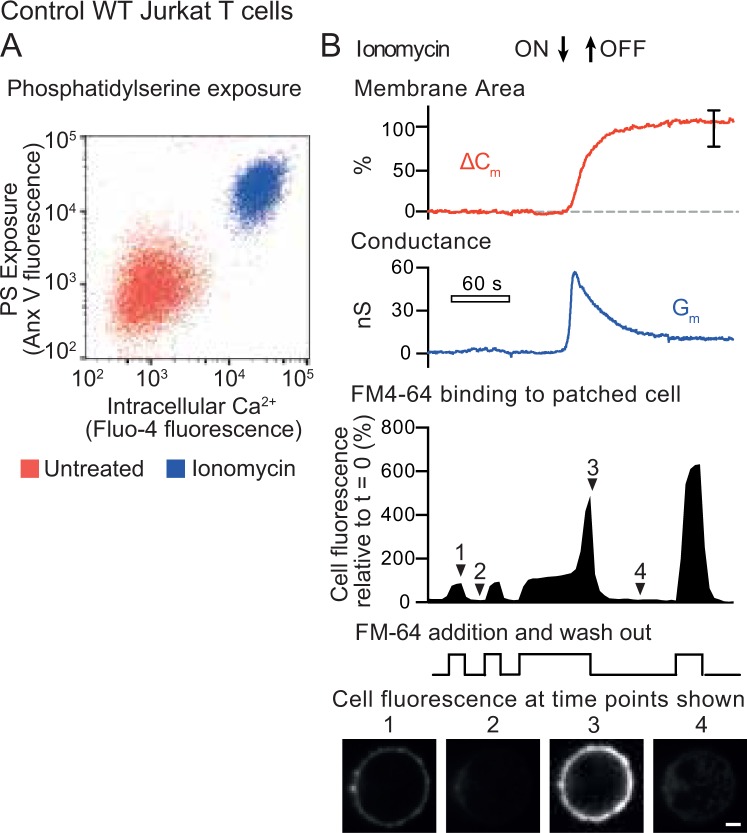
Surface phosphatidylserine exposure and plasma membrane expansion in Jurkat T cells treated with ionomycin. (**A**) Jurkat T cells were incubated with the cytoplasmic calcium indicator Fluo4-AM, then treated with 5 μM ionomycin for 15 minutes at 37 °C. Cells were then chilled on ice and stained with Annexin V (Anx V) to detect surface phosphatidylserine. Flow cytometry analysis shows intracellular calcium plotted against surface phosphatidylserine. (**B**) Single Jurkat T cells were patched with a glass micropipette loaded with cytoplasmic solution (see Materials and Methods) and incubated at 37 °C in Ringer’s solution. Cells were then treated with 5 μM ionomycin for the period shown. Measurements were made of total capacitance, C_m_, which reflects plasma membrane area, and transmembrane conductance, G_m_. The red trace shows the change in capacitance (∆C_m_) compared to t = 0, while the blue trace shows G_m_. A typical ∆C_m_ trace is shown, the error bar represents standard error of the mean (SEM) at 150 s post ionomycin addition (n = 10). In addition, the dye FM4-64, which binds reversibly to membranes, was added to the same patched cell and removed as shown. The solid trace shows total FM4-64 fluorescence of the cell, measured by a confocal microscope, relative to t = 0. Below are images taken using confocal microscopy at the time points shown (scale bar is 5 μm).

As previously reported for mouse T lymphocytes^12^, treatment of Jurkat T cells with the calcium ionphore ionomycin led to an increase in cytoplasmic Ca^2+^ (measured by Fluo-4 fluorescence) and surface exposure of PS (detected by annexin V staining) (Fig. 1A).

We had previously observed large changes in plasma membrane area when PS was exposed by Ca^2+^ ionophore treatment in BHK cells^20^. We therefore examined whether the plasma membrane (PM) area of Jurkat T cells changed when they were exposed to ionomycin. PM area was quantified by patch clamp recording of membrane capacitance (C_m_), which is proportional to membrane area. We also measured membrane conductance (G_m_) which measures the voltage-dependent movement of ions across the PM. Confocal microscopy of the patched cell was used to measure the surface binding of the fluorescent membrane dye FM4-64. During a 20 second exposure to ionomycin, C_m_ of the Jurkat T cell showed a striking doubling in plasma membrane area (Fig. 1B, C_m_ red trace), and FM4-64 dye binding also increased dramatically (Fig. 1B solid shape number 3, Fig. 1B micrograph 3, Supplementary Video S1). FM4-64 binding was fully reversible when the dye was removed, indicating that the dye was not internalised (Fig. 1B solid shape 3 to 4, Fig. 1B micrograph 4**)**. Interestingly, the PM conductance of the Jurkat T cell increased substantially and then declined in parallel with the rise in capacitance (Fig. 1B. G_m_, blue trace), consistent with the transient opening of a Ca^2+^ -gated ion channel at the time of membrane expansion.

### TMEM16F regulates cytoplasmic Ca^2+^-activated PM expansion

**Figure 2:**
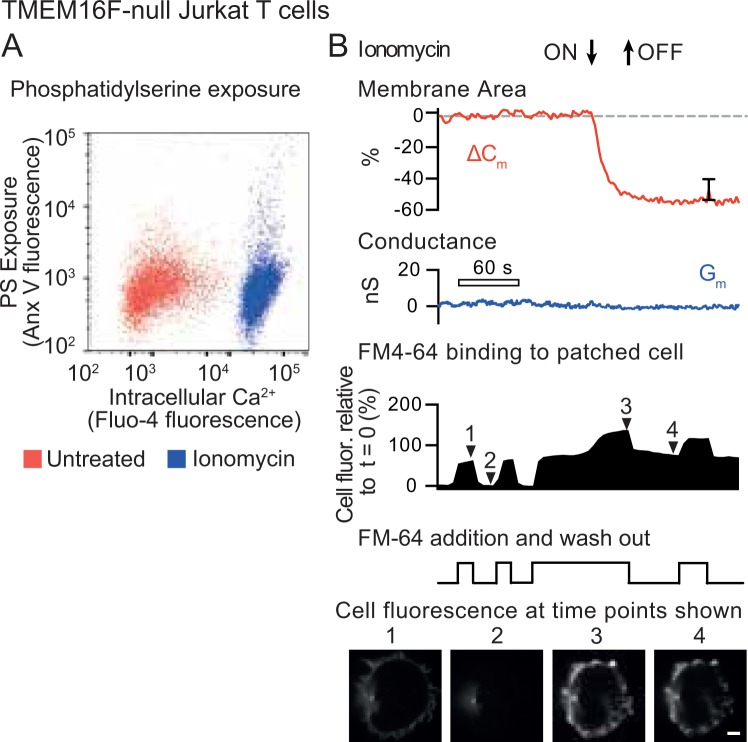
Plasma membrane internalization in TMEM16F-null Jurkat T cells treated with ionomycin. (**A**) TMEM16F-null Jurkat T cells were incubated with the cytoplasmic calcium indicator Fluo4-AM, then treated with 5 μM ionomycin for 15 minutes at 37 °C. Cells were then chilled on ice and stained with Annexin V (Anx V) to detect surface phosphatidylserine. Flow cytometry analysis shows intracellular calcium plotted against surface phosphatidylserine. (**B**) Single TMEM16F-null Jurkat T cells were patched with a glass micropipette loaded with cytoplasmic solution (see Materials and Methods) and incubated at 37 °C in Ringer’s solution. Cells were then treated with 5 μM ionomycin for the period shown. Measurements were made of total capacitance, C_m_, which reflects plasma membrane area, and transmembrane conductance, G_m_. The red trace shows the change in capacitance (∆C_m_) compared to t = 0, while the blue trace shows G_m_. A typical ∆C_m_ trace is shown, the error bar represents standard error of the mean (SEM) at 150 s post ionomycin addition (n = 10). In addition, the dye FM4-64, which binds reversibly to membranes, was added to the same patched cell and removed as shown. The solid trace shows total FM4-64 fluorescence of the cell, measured by a confocal microscope, relative to t = 0. Below are images of FM4-64 fluorescence taken using confocal microscopy at the time points shown (scale bar is 5 μm).

TMEM16F has been reported to act as a Ca^2+^-dependent phospholipid scramblase^3^ as well as a Ca^2+^ -gated ion channel^6^. We therefore tested whether our observations in Fig. 1 depended on TMEM16F by deleting it from Jurkat T cells using CRISPR/Cas9 to target TMEM16F exon 2. The sequence of exon 2 from a single cell clone isolated from the targeted cells showed three different single base pair insertions at the same position, suggesting this gene is present in three copies in Jurkat T cells (Supplementary Fig. S2C). In each case the base pair insertion was predicted to generate an in frame stop codon, truncating TMEM16F after 50 amino acids (Supplementary Fig. S2D). Antibodies against human TMEM16F did not reliably detect TMEM16F in Jurkat T cell lysates. We therefore conducted proteomic analysis of membrane preparations from wild-type and exon 2 CRISPR/Cas9 targeted cells (Supplementary Fig. S3). Data comparing TMEM16F peptides to three other representative membrane proteins demonstrate that the CRISPR/Cas9 targeted cells lack TMEM16F protein within the limits of detection. In addition, the TMEM16F-null cells failed to externalise PS in response to ionomycin despite a large increase in intracellular Ca^2+^, as previously reported for mouse T lymphocytes^12^ (Fig. 2A, Supplementary Fig. S2H). These results could be replicated in an independent cell clone derived from Jurkat T cells using CRISPR/Cas9 to target TMEM16F exon 5 (Supplementary Fig. S2E–H), the exon 2 targeted cell clone was used for all subsequent experiments.

In patch clamp recordings of Exon 2-targeted TMEM16F-null cells, the Ca^2+^-activated increase in conductance was also abolished (Fig. 2B, G_m_ blue trace), consistent with the idea that the Ca^2+^ -gated ion channel that we detected in Fig. 1B was indeed due to TMEM16F. Remarkably, in the TMEM16F-null cells PM area *decreased* by 50% upon ionomycin treatment (Fig. 2B, red trace), compared to the doubling in plasma membrane area observed in the wild-type Jurkat T cells (Fig. 1B, C_m_ red trace). This decrease in membrane area was due to PM internalisation, as FM4-64 binding became irreversible after ionomycin treatment (Fig. 2B solid shape 3 to 4) and could be detected in membrane structures below the cell surface (Fig. 2B micrograph 4, Supplementary Fig. S4. The kinetics of this endocytic response are consistent with a rapid form of Ca^2+^-activated massive endocytosis (MEND) that we have described previously in fibroblasts and that becomes activated in the presence of cytoplasmic polyamines, such as spermine and spermidine^21^. In further support of the idea that this endocytosis represents MEND, endocytic responses were not blocked by inhibiting clathrin with K^+^-free cytoplasmic solutions, or by a dynamin inhibitor, or by perturbing the actin cytoskeleton with latrunculin or phalloidin (Supplementary Fig. S5A).

The increase in intracellular Ca^2+^ triggered by ionomycin addition to Jurkat cells is actually similar to that resulting from maximal TCR triggering in this cell line^19^. However, many physiological cell stimuli and many primary cell types show smaller intracellular Ca^2+^ increases in response to receptor signalling. We therefore buffered free intracellular Ca^2+^ using EGTA and examined the effect of a more modest (3 μM) increase in free intracellular Ca^2+^. Supplementary Fig. S5B shows that this level of free intracellular Ca^2+^ causes a clear expansion in plasma membrane in the presence of TMEM16F, and substantial loss of plasma membrane when TMEM16F is absent. It is noteworthy that this level of free intracellular Ca^2+^ is substantially lower than the 100 μM free intracellular Ca^2+^ often used to studty TMEM16F function (for example)^6^, and therefore TMEM16F regulation by receptor signaling in some circumstances remains likely.

### Ca^2+^-activated PS exposure and PM expansion is followed by vesicle shedding

**Figure 3:**
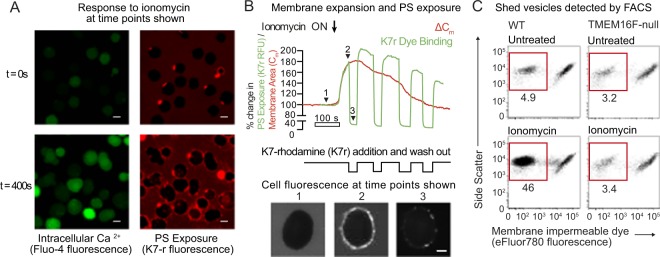
Simultaneous surface phosphatidylserine exposure and plasma membrane expansion is followed by membrane vesicle shedding. (**A**) Jurkat T cells were loaded with the cytoplasmic calcium indicator Fluo4-AM, then treated with 5 μM ionomycin for 400 s at 37 °C in the presence of polylysine-rhodamine (K7r) which binds rapidly to exposed phosphatidylserine. Confocal microscope images of Fluo4-AM and K7r fluorescence in a single field of cells are shown (scale bar is 10 μm). (**B**) A single TMEM16F-null Jurkat T cells was patched with a glass micropipette loaded with cytoplasmic solution (see Materials and Methods), incubated at 37 °C in Ringer’s solution, then treated with 5 μM ionomycin at the time shown. Total capacitance, C_m_, was measured and the red trace shows the change in capacitance (∆C_m_) compared to t = 0. In addition, K7r was added to the same patched cell and removed as shown. The green trace shows total K7r fluorescence of the cell, measured by a confocal microscope, relative to t = 0. Below are images of K7r fluorescence taken using confocal microscopy at the time points shown (scale bar is 5 μm). **(C)** Wild-type or TMEM16F-null Jurkat T cells were treated with 5 μM ionomycin for 15 minutes in the presence of eFluor780, a plasma membrane impermeable dye. The cell preparations were analyzed by FACS. The events shown were first gated on subcellular sized particles using forward scatter and side scatter. Staining of these subcellular particles is displayed versus side scatter, with unstained events representing dye impermeable membrane vesicles rather than apoptotic bodies. The numbers shown represent the percentage of total events.

We next investigated the timing of PS exposure and membrane expansion after ionomycin treatment. To detect PS exposure, we used a rhodamine labelled cationic peptide, heptalysinerhodamine (K7r), rather than annexin V. K7r binds anionic phospholipids more rapidly than annexin V and does not require Ca^2+^-containing buffers for binding^20^. Figure 3A illustrates imaging of a single field of Jurkat T cells, measuring cytoplasmic Ca^2+^ using Fluo-4-AM (green), and PS exposure (red) using K7r. Treatment with ionomycin resulted in increases in cytoplasmic Fluo-4 fluorescence followed after 45 seconds by PM labelling with K7r. The full video of this experiment with unpatched cells can be seen in Supplementary Video S6. Figure 3B shows parallel measurements of the increase in membrane expansion, C_m_ (red trace), and PS exposure, K7r (green trace) in a single patch-clamped Jurkat T cell. The two signals followed an identical time course within the detection limits of the experiment, suggesting that PS exposure and PM expansion are linked processes. Subsequent to PM expansion and PS exposure, the two fluorescence signals declined in parallel. The fact that the K7r signal was not maintained as C_m_ declined, (Fig. 3B), suggested that the excess PM was shed rather than endocytosed. Vesicle shedding preceeded by membrane protrusions could be detected when FM4-64-stained Jurkat T cells were treated with ionomycin and followed by video microscopy (Supplementary Video S1).

In order to follow the fate of this shed membrane we treated wild-type and TMEM16F-null Jurkat T cells with ionomycin for 10 minutes, then stained the cultures with Fluor®780, a membrane impermeable dye. Flow cytometry was then used to analyse subcellular-sized events, which were separated into those with intact membranes, or those with ruptured membranes by Fluor®780 staining (Fig. 3C). All cultures contained similar levels of subcellular, membrane permeable particles. However, Ca^2+^-ionophore treatment of WT but not TMEM16F-null Jurkat T cells resulted in a large increase of subcellular events with an intact membrane, consistent with a TMEM16F-dependent release of membrane vesicles.

### Relationship between TMEM16F cation conduction and PM expansion

**Figure 4:**
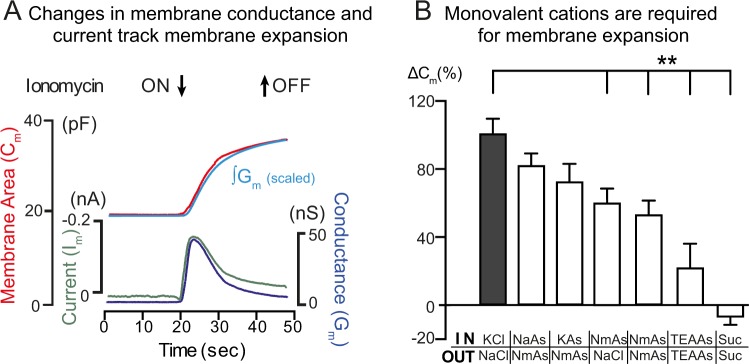
Membrane expansion occurs in parallel with conductance and current changes and requires the presence of monovalent cations. (**A**) Single Jurkat T cells were patched with a glass micropipette loaded with cytoplasmic solution (see Materials and Methods) and incubated at 37 °C in Ringer’s solution. Cells were treated with 5 μM ionomycin for the period shown. Measurements were made of total capacitance, C_m_, transmembrane conductance, G_m_ (lower blue trace), and transmembrane current I_m_ (lower green trace). The upper red trace shows the change in capacitance (∆C_m_), while the upper blue trace shows the integral ∫G_m_. Records were scaled to start and end at the same time, n = 10. (**B**) Membrane expansion was recorded as in ‘A’ and monovalent cation concentrations employed were varied as indicated.

TMEM16F is also reported to act as a non-selective cation channel^6^. We therefore investigated the relationship between monovalent cation transport and PM expansion during TMEM16F activation. Figure 4 shows the result for patched Jurkat T cells in which PM expansion was monitored using capacitance (C_m_) changes,. Membrane ion current (I_m_) and conductance (G_m_) were also measured. Cytoplasmic Ca^2+^ elevation was induced by ionomycin treatment. As shown in Fig. 4A, the time course of increase in C_m_ closely followed the time courses of membrane current (I_m_) and conductance (G_m_) changes. The integral of the G_m_ signal corresponds closely to the time course of the C_m_ signal, after scaling (>20 observations). This suggests that PM expansion is directly dependent on the activation state of TMEM16F. Expansion begins as TMEM16F channels open and terminates as TMEM16F channels inactivate.

Given this close relationship, we next tested whether monovalent cations that permeate TMEM16F affect PM expansion (Fig. 4B). To do so, we varied the monovalent content of experimental solutions systematically. From left to right, the results shown that substitution of >90% of Cl for aspartate (As) was without effect, that both cytoplasmic K and extracellular Na could support extensive PM expansion in the presence of NMDG (Nm) on the opposite membrane side, that that presence of Nm on both membrane sides reduced expansion by ~60%, that the nearly impermeable cation, TEA, reduced expansion by ~75%, and that substitution of 90% of monovalent ions for sucrose abolished membrane expansion. While these data demonstrate that membrane expansion requires monovalent cations, the ability of both cytoplasmic K and extracellular Na to support expansion excludes the idea that either cation efflux or influx catalyzes membrane expansion.

### TMEM16F mutations modulate its function

**Figure 5:**
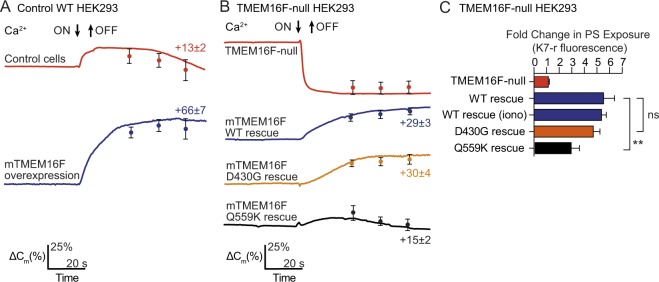
Plasma membrane expansion activity of TMEM16F mutants (**A**) Single HEK293 cells stably expressing the cardiac Na/Ca exchanger (NCX1.1) were patched with a glass micropipette loaded with 40 mM Na^+^; transient increase of extracellular [Ca^2+^] to 2 mM (at the time shown) was used to induce an increase in intracellular Ca^2+^. The change in membrane capacitance (∆C_m_) was measured and the percentage change was calculated. Single recordings are displayed, mean values at 30 s, 45 s and 60 s after stimulation are shown (+/−SEM, n = 7). Upper red trace, control cells; lower blue trace, cells over-expressing murine TMEM16F. (**B**) Single TMEM16F-null HEK293 cells stably expressing the cardiac Na/Ca exchanger (NCX1.1) were patched with a glass micropipette loaded with 40 mM Na^+^; transient increase of extracellular [Ca^2+^] to 2 mM (at the time shown) was used to induce an increase in intracellular Ca^2+^. The change in membrane capacitance (∆C_m_) was measured and the percentage change was calculated. Single recordings are displayed with mean values at 30 s, 45 s and 60 s after stimulation shown (+/−SEM, n = 7). Upper red trace, control cells; lower traces, cells over-expressing murine TMEM16F and mutants as shown. (**C**) Single TMEM16F-null HEK293 cells stably expressing the cardiac Na/Ca exchanger (NCX1.1) were patched with a glass micropipette loaded with 40 mM Na^+^; a transient increase of extracellular [Ca^2+^] to 2 mM was used to induce an increase in intracellular Ca^2+^ in the presence of the phosphatidylserine binding dye K7r. In the case of “WT rescue (iono)” cells were treated with 5 μM ionomycin instead. The total fluorescence of each cell was measured at 60 s using a confocal microscope, and the percentage change compared to untreated was calculated. Mean values are shown (+/−SEM, n = 7; **p < 0.01, ns, not significant, Student t-test).

We next examined the activity of TMEM16F mutants that are reported to have modified scrambling activity^3,6,22^. In these experiments we used patch clamped HEK 293 cells that constitutively overexpress the NCX1 Na^+^/Ca^2+^ exchanger^20^. This allows the generation of a transient increase in cytoplasmic Ca^2+^, by elevating external Ca^2+^, without the use of ionomycin. Figure 5A (red trace) shows that these HEK 293 cells also responded to a cytoplasmic Ca^2+^ increase with a modest PM expansion, an effect that was augmented by over-expression of murine TMEM16F (mTMEM16F) (Fig. 5A, blue trace).

We then used the same CRISPR/Cas9 targeting exon 2 to create a TMEM16F-null HEK 293 cell line overexpressing the NCX1 Na/Ca exchanger. As in Jurkat cells, Ca^2+^ influx in TMEM16F-null HEK293 cells caused rapid MEND (Fig. 5B, red trace), and both membrane expansion (Fig. 5B, blue trace) and PS exposure induced by either increasing extracellular [Ca^2+^] or addition of ionomycin (Fig. 5C, blue bars) could be restored by over-expressing mTMEM16F. The D430G mutant was reported to have constitutive PS scrambling activity^3^, however, we saw no difference in PS exposure between mTMEM16F and the D430G mutant, in agreement with a more recent report^6^ (Fig. 5C, orange bar). In line with this result, we further demonstrated no difference in membrane expansion by the D430G mutant (Fig. 5B, orange trace**)**. However the Q559K TMEM16F mutantion, which alters ion selectivity and slows activation kinetics^6^, had diminished PS exposure (Fig. 5C, black bar). Notably, however, the residual function of the Q559K mutant was sufficient to prevent the MEND response seen in TMEM16F-null cells (Fig. 5B, black trace).

We have previously reported that polyamines, which can modulate the activity of ion channels^23^, can switch Ca^2+^-induced membrane expansion to fast MEND responses in fibroblasts^21^, reminiscent of the effects of deleting TMEM16F. As shown in Fig. S7, spermine at physiological concentrations (up to 500 μM) also changed the ionomycin-induced Jurkat T cell PM expansion to MEND. The TMEM16F conductance was also blocked by spermine. This might reflect either a direct block of the TMEM16F pore or binding of anionic lipids that appear to regulate TMEM16F activity as recently demonstrated for PIP_2_^24^. Given that TMEM16F conductance invariably decreases as PS is lost from cytoplasmic leaflette, we suggest that cytoplasmic PS may also support channel activity. As in the case of the Q559K mutant of TMEM16F, an intermediate concentration of spermine inhibited membrane expansion and did not lead to MEND in response to ionomycin.

### PD-1 is shed in vesicles after Ca^2+^-activated PM expansion

**Figure 6:**
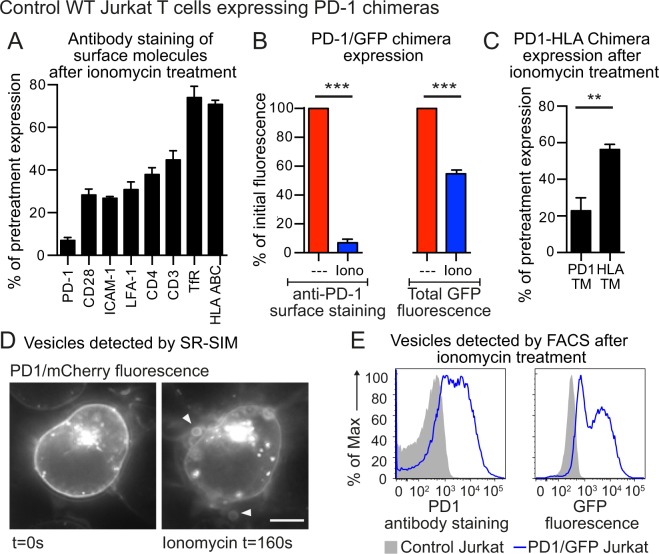
Wild-type Jurkat T cells shed PD-1 upon treatment with ionomycin (**A**) Control wild-type Jurkat T cells over-expressing a PD-1/GFP chimera were treated with 5 μM ionomycin for 15 minutes at 37 °C for 15 minutes then chilled and stained at 4 °C for expression of a variety of surface molecules and analyzed by FACS. Data shows the percentage of initial surface expression after ionomycin treatment. (**B**) Control wild-type Jurkat T cells over-expressing a PD-1/GFP chimera were treated with 5 μM ionomycin for 15 minutes at 37 °C then chilled and stained at 4 °C for PD-1 expression. PD-1 staining and cellular GFP expression were analyzed by FACS; data shows the percentage of initial expression after ionomycin treatment. (**C**) Control wild-type Jurkat T cells over-expressing a PD-1/GFP chimera, or a PD-1/GFP chimera where the PD-1 transmembrane sequence was replaced by that from HLA-A2, were treated with 5 μM ionomycin for 15 minutes at 37 °C then chilled and stained at 4 °C for PD-1 expression. PD-1 staining was analyzed by FACS; data shows the percentage of initial expression after ionomycin treatment. **(D)** Control wild-type Jurkat T cells over-expressing a PD-1/mCherry chimera, were imaged by SR-SIM microscopy before and after ionomycin treatment. Arrows indicate fluorescent vesicles (scale bar is 5 μm). (**E**) Control wild-type Jurkat T cells, or control wild-type Jurkat T cells over-expressing a PD-1/GFP chimera, were stained with an anti-PD-1 antibody, then treated with 5 μM ionomycin for 15 minutes at 37 °C in the presence of fixable viability dye eFluor780. The cell preparations were analyzed by FACS. The events shown were first gated on subcellular sized events (low forward scatter) and intact membranes (low fixable viability dye eFluor780 staining). PD-1 and GFP expression in these gated events is shown. Cellular FACS data shows the mean +/− SEM for three independent experiments. (***p < 0.001, **p < 0.01, Student t-test).

We next investigated the surface expression of T cell proteins following Ca^2+^-ionophore treatment. Initially, we examined the regulation of PD-1, as TMEM16F has been implicated in control of PD-1 surface expression^12^. PD-1 was expressed only at low levels in Jurkat T cells so we upregulated irs expression with PHA and showed that ionophore treatment resulted in rapid PD-1 down-regulation (Fig. S8). The same effect could be seen in PBML, where OKT3 upregulated PD-1 on a subset of cells. This up-regulated PD-1 was rapidly down-regulated by ionomycin treatment (Fig. S8). To track PD-1 during membrane expansion and shedding, we engineered Jurkat T cells to constitutively express a PD-1/GFP chimera. Figure 6A shows that both endogenous and chimeric PD-1 were the most down-regulated surface proteins among those that we studied, including the co-receptor CD28, the adhesion molecules ICAM-1 and LFA-1, and the highly expressed transferrin receptor and HLA-A, -B and -C. We then used the PD-1/GFP fusion protein to analyse the cause of this decrease in surface expression. Figure 6B shows that approximately 50% of the PD-1/GFP fluorescence was lost from the cells on ionophore treatment. We reasoned that the extent of PD-1/GFP loss was not as great as the loss of PD-1 surface staining because some of the PD-1/GFP was present intracellularly (as demonstrated by PD-1/mCherry fluorescence, used for compatibility with the microscope, in Fig. 6D). In order to examine which part of the PD-1 molecule controlled shedding, we constructed chimeras between PD-1 and HLA-A2, as HLA staining did not change significantly in response to ionophore treatment (Fig. 6A). The most informative chimeras are shown in Fig. 6C, both proteins have the PD-1 extracellular domain and the HLA-A2 intracellular domain while one had the PD-1 transmembrane domain (PD1TM), while the other had the HLA-A2 transmembrane domain (HLATM). Figure 6C shows that the transmembrane domain of PD-1 was necessary for its down-regulation after ionophore treatment.

We then investigated whether PD-1 was shed in vesicles, using both microscopy and flow cytometry. In order to permit detection by Structured Illumination Microscopy (SR-SIM), we used a PD-1/mCherry fusion protein, as opposed to PD-1/GFP for compatibility with the microscope. Figure 6D shows that we could detect fluorescent vesicles after ionomycin treatment and Supplementary Fig. S9 is a video of this experiment. When we used flow cytometry to detect subcellular sized events with intact membranes we found vesicles containing PD-1, detected by both antibody staining and GFP fluorescence, in the supernatant of cells 15 minutes after ionomycin treatment (Fig. 6E).

### PD-1 is internalized in cells lacking TMEM16F

**Figure 7:**
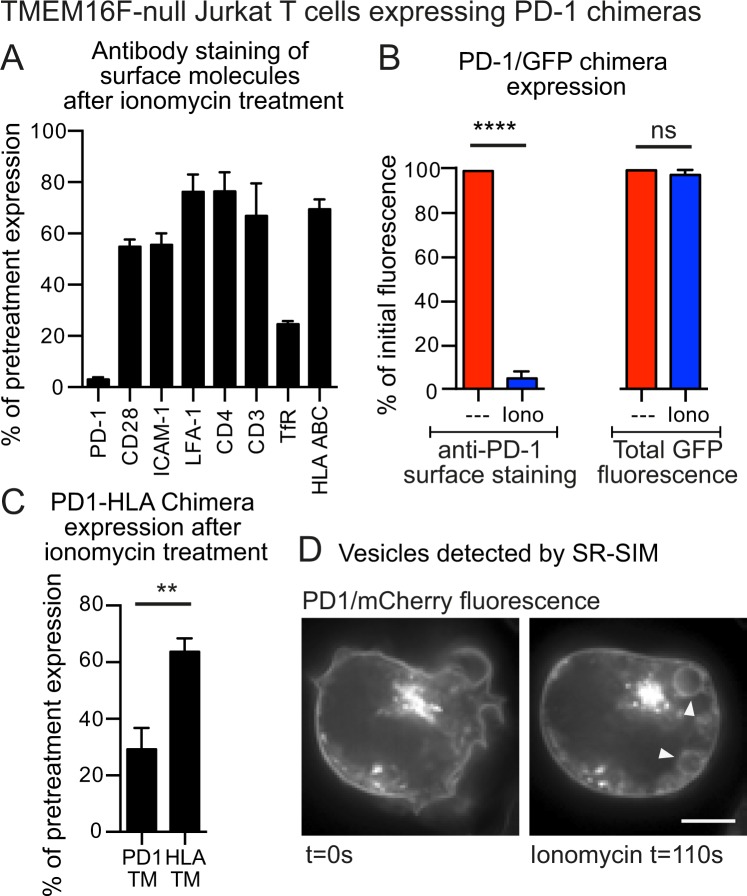
TMEM16F-null Jurkat T cells internalise PD-1 upon treatment with ionomycin (**A**) TMEM16F-null Jurkat T cells over-expressing a PD-1/GFP chimera were treated with 5 μM ionomycin for 15 minutes at 37 °C for 15 minutes then chilled and stained at 4 °C for expression of a variety of surface molecules and analyzed by FACS. Data shows the percentage of initial surface expression after ionomycin treatment. (**B**) TMEM16F-null Jurkat T cells over-expressing a PD-1/GFP chimera were treated with 5 μM ionomycin for 15 minutes at 37 °C then chilled and stained at 4 °C for PD-1 expression. PD-1 staining and cellular GFP expression were analyzed by FACS; data shows the percentage of initial expression after ionomycin treatment. (**C**) TMEM16F-null Jurkat T cells over-expressing a PD-1/GFP chimera, or a PD-1/GFP chimera where the PD-1 transmembrane sequence was replaced by that from HLA-A2, were treated with 5 μM ionomycin for 15 minutes at 37 °C then chilled and stained at 4 °C for PD-1 expression. PD-1 staining was analyzed by FACS; data shows the percentage of initial expression after ionomycin treatment. (**D**) TMEM16F-null Jurkat T cells over-expressing a PD-1/mCherry chimera, were imaged by SR-SIM microscopy before and after ionomycin treatment. Arrows indicate fluorescent vesicles (scale bar is 5 μm). Cellular FACS data shows the mean +/− SEM for three independent experiments. (****p < 0.0001, **p < 0.01, Student t-test).

We next examined PD-1 expression in TMEM16F-null cells using antibody staining and found that this decreased substantially following ionomycin treatment (Fig. 7A). In this case, however, the PD-1/GFP chimera was not lost from the cells when GFP fluorescence was measured after ionomycin treatment, though it disappeared from the cell surface suggesting that it had been internalized, presumably by endocytosis (Fig. 7B). Comparison of the PD1 and HLA-A2 chimeras demonstrated that the PD-1 transmembrane domain was required for PD-1 down-regulation in response to ionomycin (Fig. 7C). Finally, the PD-1/mCherry chimera could be found in large intracellular vesicles after ionophore treatment (Fig. 7D, Supplementary Video S10) suggesting that that it is internalized by endocytosis during MEND in TMEM16F-null cells.

## Discussion

### How are TMEM16F-induced membrane expansion, PS exposure and ion conduction related?

These data demonstrate that TMEM16F can act as a cytoplasmic Ca^2+^ sensor/effector for large-scale membrane expansion (Figs 1 and 3–5), in addition to its previously known functions as a phospholipid scramblase and an ion channel. Membrane expansion closely followed the kinetics of PS exposure (Fig. 3), suggesting interdependence of these activities. Significantly, membrane expansion activity was impaired by a mutation (Q559K) that affects the ion selectivity, activation kinetics and phospholipid scrambling activity of TMEM16F (Fig. 5)^6,22^. The mechanism of large-scale membrane expansion is not at all clear from these data. The major possibility raised is that phospholipid scrambling in some way triggers PM expansion, where by loss of cytoplasmic PS or a gain of extracellular PS might play critical roles. The present data also do not exclude a possibility that TMEM16F-activated PS exposure reflects the fusion of intracellular membranes that have randomised phospholipids^25,26^. The fact that both cytoplasmic K and extracellular Na can support membrane expansion (Fig. 4) rules out the possibilies that cation efflux or cation influx per se might triggers PM expansion by changing local cation concentrations. Rather, cations must be playing some other structural or catalytic roles in the PM expansion process, possibly related to phospholipid scrambling.

The identity of the membranes that fuse with the cell surface, and whether this occurs by canonical exocytosis or by some other mechanism, remain unanswered questions from the present results. Compartments that may in principle be involved include lysosomes, ER, recycling endosomes, Golgi-derived tubules^25,27^, and a poorly understood membrane compartment dubbed ‘enlargeosomes’ in previous studies^28^. Further investigation is clearly essential to resolve both the mechanistic and structural basis of TMEM16F-dependent PM expansion.

### How does TMEM16F prevent endocytosis?

We were surprised to find that the presence or absence of TMEM16F determines whether large amounts membrane are added to or removed from the PM in response to an increase in cytoplasmic Ca^2+^ (Figs 1 and 2). It appears that TMEM16F blocks endocytosis in response to Ca^2+^ influx via ionphore addition, because the TMEM16F mutant Q559K (Fig. 5) showed neither PM expansion nor endocytosis.This suggests that a minimally active TMEM16F is sufficient to block endocytosis. Furthermore, the presence of a polyamine, spermine, that blocks TMEM16F conductance also blocks PM expansion and promotes endocytosis (Supplementary Fig. S7). Similar results obtained for spermidine in BHK cells supports the idea that block of TMEM16F activity supports endocytosis by nonconventional mechanisms^21^. For example, it may be that TMEM16F-induced phospholipid scrambling promotes membrane disorder, shown to inhibit MEND^29^. Alternatively cytoplasmic PS may be required for endocytosis, and its loss via TMEM16F-mediated scrambling accordingly blocks this form of fast Ca^2+^-activated MEND.

### What is the role of TMEM16F in membrane shedding and PD-1 regulation?

We have described that TMEM16F-dependent PM expansion leads to extensive shedding of membrane vesicles from the PM as ectosomes (Fig. 3). Previous reports demonstrate that TMEM16F-null erythrocytes do not produce vesicles in response to a Ca^2+^ signal^30^. Bevers and Williamson have proposed that PS-expressing vesicles act as a catalyst for bone mineralization^31^ which would explain why TMEM16F-null mice have defective embryonic bone formation: they are unable to produce such vesicles^32^.

In the case of T cells, the shed membrane vesicles express the immune inhibitor PD-1, which would account for the reduction of PD-1 on the cell surface (Fig. 7). Hu and colleagues^12^ found that TMEM16F-deficient T cells exhibited high PD-1 expression. This was accompanied by immune exhaustion and impaired anti-viral immunity. They detected TMEM16F in endosomes at the immunological synapse in T cells, and suggested that TMEM16F limits PD-1 surface expression, by causing its endocytosis. To the contrary, our data suggest that it is the shedding of PD-1 that accounts for its down-regulation. The fact that TMEM16F-deficient T cells exhibited high PD-1 expression also leads to the conclusion that down-regulation of PD-1 by MEND does not occur in mice, perhaps because of other known defects in TCR signalling in these exhausted cells^12^. The observation that the PD-1 transmembrane region controls its association with membrane that is either shed in vesicles or endocytosed (Figs 6 and 7) suggests that particular transmembrane domains must be involved in cytoplasmic Ca^2+^-stimulated trafficking.

Vesicle shedding from T cells can be a localised phenomenon following TCR triggering^33^. Our study is limited in that we have used a T cell line that is amenable to patch-clamp experiments, and a non-physiological Ca^2+^-ionophore stimulus to induce whole cell responses so that we can measure bulk plasma membrane changes using capacitance. Future studies of the role of TMEM16F measuring local vesicle shedding in primary T cells following receptor triggering are therefore warranted.

### What is the role of TMEM16F in membrane repair?

It has long been recognised that cells can rapidly repair mechanical damage to their PM^34^. This is one of the processes that we are mimicking when we induce a large increase of cytoplasmic Ca^2+^ using an ionophore. TMEM16F has a high Ca^2+^ threshold for activation^4,6^ and membrane trafficking events triggered by similarly high Ca^2+^ levels occur during membrane repair. A physical lesion or a pore-forming toxin increases the cytoplasmic Ca^2+^ concentration by up to a hundred-fold, which has been reported to cause exocytosis^35^, endocytosis^20,36^, and/or membrane shedding^37,38^. Thus one potential function of Ca^2+^-stimulated exocytosis dependent on TMEM16F may be membrane repair, with the expression level or activity of TMEM16F controlling whether cells respond by exocytosis or endocytosis. Indeed, physiological membrane damage and repair in animals have been most extensively studied in muscle^35^ where mechanical damage induces Ca^2+^-dependent membrane repair. Deficiency in TMEM16E, a close family member of TMEM16F, causes a muscular dystrophy that is linked to defective membrane fusion and repair^39^. It remains to be established whether TMEM16F is involved in membrane repair in other cell types.

## Materials and Methods

Data analysis was performed in Prism (GraphPad) or SigmaPlot (Systat) unless otherwise stated.

### Cell lines, solutions and reagents

Jurkat E6.1 T cells (ECACC) were grown in RPMI-1640 (Sigma-Aldrich) while HEK-293 cells (Invitrogen) were grown in DMEM (Sigma-Aldrich). All media were supplemented with 10% FCS (Invitrogen), 2 mM L-glutamine (Invitrogen) with 100 U/mL penicillin and 100 µg/mL streptomycin (Invitrogen). Standard cytoplasmic solution (Figs 1–5) contained (in mM) 145 KCl, 10 HEPES, 0.5 EGTA, 0.25 CaCl_2_, 8 MgATP, 2 TrisATP and 0.2 GTP, adjusted to pH 7.4. Extracellular ‘Ringer’ solution (Figs 1–5) contained (in mM) 145 NaCl, 10 HEPES, 2 CaCl_2_ and 2 MgCl_2_, adjusted to pH 7. Intracellular solution used in Fig. 4B contained (in mM) 100 NMDG, 40 NaOH, 140 aspartic acid, 0.5 EGTA, 0.25 CaCl_2_, 0.5 MgCl_2_, 10 HEPES adjusted to pH 7.0. K^+^-free *N*-Methyl-D-glucamine aspartate solution (Fig. S5) contained (in mM) 145 NMDG, 10 HEPES, and 0.5 EGTA, adjusted to pH 7 with aspartic acid; 2 CaCl_2_ or 2 MgCl_2_ were added when solution was used on the extracellular side while 0.25 and 0.5, resp., were used on the cytoplasmic side. 5 μM ionomycin free acid (Calbiochem) was used unless otherwise stated. Other reagents used were: Lipofectamine 3000 (Life Technologies) for transient transfection protocols; 5 μM FM 4-64 (Life Technologies), and 50–500 μM spermine (Sigma-Aldrich). Rhodamine-conjugated hepta-lysine (rhodamine-KKKKKKK-amide; K7r) was prepared by Multiple Peptide Systems (NeoMPS, Inc.) and used at 3 μM. Compared to Annexin V, K7r was advantageous for real-time imaging as membrane binding is significantly faster, and K7r binding does not require Ca^2+^.

### Whole-cell patch clamp with confocal imaging

Patch clamp recordings of cell electrical parameters were performed as described previously^20^ using Capmeter v6.3^40^ with National Instruments digital acquisition boards and Axopatch 200B or 1D patch clamp amplifiers. Square-wave voltage perturbation (20 mV; 0.5 kHz) was employed, input resistances were 2–9 MΩ, and the apparent cell resistances were 0.1–2 GΩ. External solutions were kept at 35–37 °C in parallel gravity-fed solution lines with outlet flow rates of 2 to 5 mm/s. When confocal imaging and electrophysiology were combined, a Nikon TE2000-U microscope; 60 × oil immersion, 1.45-NA objective paired with the EZ-C1confocal system (Nikon) containing a 40-mW 163-Argon laser (Spectra Physics; Newport Corporation) operating at 488 nm at 7.5% maximum capacity for FM4-64 recordings and a 1.5 mW Melles Griot cylindrical HeNe laser at 543 nm at 55% of maximum capacity for K7r. Emission filters were set to either 500–540 nm or 580LP. Typical resolution was 512 × 512, set with a pixel dwell time yielding <1-s exposure times per frame and a pinhole of 150 μm. Image analysis was performed using either the EZ-C1 v3.9 (Nikon Instruments) or ImageJ (NIH). When imaging multiple fluorophores, sequential imaging was used to minimize spectral overlap. Photobleaching was negligible for these experiments. For independent confocal imaging of intact cells, 800 μl of media containing Jurkat cells was transferred to a glass bottom 4-chamber dish. Warmed ionomycin solution was rapidly added at a 5:1 concentration and cells were imaged as described. For patch clamp, intracellular calcium stimulus was triggered by direct perfusion of ionomycin for Jurkat and HEK cell lines. HEK cells stably expressing the cardiac Na/Ca exchanger (NCX1.1) were stimulated through reverse transport by cytoplasmic pipette perfusion of cells with 40 mM Na^+^ and transient application of 2 mM Ca^2+^ to the extracellular solution^20^. No difference was detected in ionomycin or NXC induced stimulation of HEK cells (Fig. 5C).

### CRISPR-Cas9 knockdown of TMEM16F

The lentiCRISPR v2 plasmid was used as described^41^. The guideRNA sequence targeting TMEM16F Exon 2 was 5′-TCAGCATGATTTTCGAACCC-3′. HEK-293 cells were transiently transfected with plasmid using Lipofectamine (Life Technologies) and the lentivirus was collected from the supernatant. Jurkat T cells and HEK-293 cells were transduced with lentivirus produced using the gag-pol expression vector p8.91 and VSV-G envelope vector pMDG as described previously^42^. 48 hours after transduction cells were selected in 0.5 μg/ml Puromycin for Jurkat and 5 μg/ml Puromycin for HEK-293 before single-cell clones were isolated. Cells stably expressing Cas-9 and gRNA after transduction were frozen after initial expansion and aliquots thawed as needed to avoid accrued off-target effects resulting from long-term culture. Gene editing was confirmed by sequencing (Beckman Coulter) (Fig. S2). Two different primer sets were used to amplify Exon 2 (Set 1 and 2). Primer sequences were as follows (all 5′ - 3′):

Set 1 FW GTGCAGGTTCATGCTTCATTT,

Set 2 FW CCCGGTGCTGCTGATTTA,

Set 3 FW AGTGGTGGTCTCTGTATTGTTT

### Proteomic analysis of Jurkat WT and CRISPR-Cas9 knock-out cells

Cells were subjected to Dounce homogenation on ice in 250 mM sucrose/10 mM Tris pH 7.4 with cOmplete protease inhibitor (Roche). Cell debris was pelleted and the supernatant was spun at 2000 g for 60 min to pellet membrane. Membranes were washed and reconstituted with 4 X Laemmli buffer for SDS-PAGE. Protein bands were excised and digested overnight with trypsin (Pierce) following reduction and alkylation with DTT and iodoacetamide (Sigma-Aldrich). The samples underwent solid-phase extraction cleanup with Oasis HLB plates (Waters) and the resulting samples were analyzed by LC/MS/MS, using an Orbitrap Fusion Lumos mass spectrometer (Thermo Electron) coupled to an Ultimate 3000 RSLC-Nano liquid chromatography systems (Dionex). Raw MS data files were converted to a peak list format and analyzed using the central proteomics facilities pipeline (CPFP), version 2.0.3^43,44^. Peptide identification was performed using the X!Tandem^45^ and open MS search algorithm (OMSSA) search engines against the human protein database from Uniprot, with common contaminants and reversed decoy sequences appended^46^. Label-free quantitation of proteins across samples was performed using SINQ normalized spectral index Software^47^.

### Chimeric proteins, lentiviral vector constructs and production

All chimeric proteins were produced using overlap-extension PCR^48^ and cloned into a single (pSIN) or dual (pDUAL) promoter vector. In fluorescent constructs a ‘GS’ linker separated PD-1 and fluorophore. Primers used to make PD1/GFP were PD1-FW, PD1GFP Sense, and PD1GFP Antisense GFP-RS. PD-1 mCherry was made in the same way using the primers PD1FW, PD1mCherry Sense, PD1mCherry Antisense and mCherry RS. PD1HLA Chimers were made with PD1-FW and HLA-A2-RS. For PD-1HLA-A2 chimeras, the extracellular portion was always PD-1 and the intracellular HLA-A2. The TM regions were the only sequences to vary between the two constructs. The transmembrane regions were predicted using the TMPred algorithm (https://embnet.vital-it.ch/software/TMPRED_form.html. The PD-1 TM was predicted as L168 to I191 and HLA-A2 TM I410 to W435. The chimera with PD-1 transmembrane was made with PD1TM Sense and PD1TM Antisense primers. The chimera with HLA-A2 transmembrane was made with HLATM Sense and HLATM Antisense primer. Lentivirus was produced using the gag-pol expression vector p8.91 and VSV-G envelope vector pMDG as described previously. The primer sequences were as follows:

PD1-FW GGGGGGATCCGCCACCATGCAGATCCCACAGGCGCC,

PD1GFP Sense GGCTCCGGCTCCGGCTCCGTGAGCAAGGGCGAGGAGCT,

PD1GFP Antisense GGAGCCGGAGCCGGAGCCGAGGGGCCAAGAGCAGTGTC,

GFP-RS GCGGCCGCTTTACTTGTACAGCTCGTCCATGCCGAGAGTGATCCC,

PD1mCherry Sense GGCTCCGGCTCCGGCTCCGTGAGCAAGGGCGAGGAGGA,

PD1mCherry Antisense GACACTGCTCTTGGCCCCTCGGCTCCGGCTCCGGCTCC,

mCherry RS GGCTCCGGCTCCGGCTCCGTGAGCAAGGGCGAGGAGGA,

HLA-A2-RS CCCCGCGGCCGCTCACACTTTACAAGCTGTGAGAGACACATCAG,

PD1TM Sense TCTGGGTCCTGGCCGTCATCAGGAGGAAGAGCTCAGATAG,

PD1TM Antisense CTATCTGAGCTCTTCCTCCTGATGACGGCCAGGACCCAGA,

HLA TM Sense CAGCCGGCCAGTTCCAAACCATCGTGGGCATCATTGCTGG,

HLA TM Antisense CCAGCAATGATGCCCACGATGGTTTGGAACTGGCCGGCTG.

### Super-resolution structured illumination microscopy (SR-SIM) and Zeiss Airyscan microscopy

SR-SIM and Airyscan imaging was performed at 37 °C using Plan-Apochromat 63×/1.4 oil DIC M27 objective in an Elyra PS.1 microscope (Zeiss). For SR-SIM, images were acquired using 5 phase shifts and 3 grid rotations, with 34 µm grating period for the 561 nm laser and filter set 4 (1851-248, Zeiss). Airyscan z-stacks were obtained at 0.75 μm steps for a total depth of 12 μm. Stacks were obtained every 30 sec using a fast piezo controller and Definite Focus 2.0 (Zeiss) to avoid z-drift between time points. Processed images were projected into a single 2-D image based on maximum intensity using ImageJ. SR-SIM images were acquired using an sCMOS camera and high-resolution Airyscan images were acquired using the LS880 GaAsP detector, both processed using ZEN software (Zeiss).

### Flow cytometry, antibodies and dyes

Cells were treated with ionomycin for 15 min at 37 °C in Ringer solution containing 2 mM CaCl_2_. Cells were stained for 30 min on ice and flow cytometry was performed using a BD Fortessa (BD Bioscience). Intracellular Ca^2+^ concentration was monitored with 1 μM Fluo-4 AM (Life Technologies) loaded in cells for 30 min at 37 °C prior to the experiment. Surface exposure of phosphatidylserine was detected using 9 ng/ml Annexin V staining in Annexin binding buffer (BioLegend). Microvesicles were distinguished from apoptotic bodies using the fixable viability dye eFluor780 dye (eBioscience). Surface staining for receptors was carried out for 30 min on ice using the following antibodies: PD-1 APC (MIH4), ICAM-1 PE (HA58), Transferrin receptor (TfR) PE (M-A712) from BD Biosciences and CD28 PE (CD28.2), LFA-1 PE (HI111), CD4 PE (OKT4), CD3 PE/Cy7 (UCHT1), HLA ABC PE (W6/32) from BioLegend. Data were analyzed using FlowJo Software (Treestar).

## Supplementary information

Supplementary data

Supplementary video S1

Supplementary video S6

Supplementary video S9

Supplementary video S10

## References

[1] Leventis PA, Grinstein S (2010). The distribution and function of phosphatidylserine in cellular membranes. Annual review of biophysics.

[2] Nagata S, Suzuki J, Segawa K, Fujii T (2016). Exposure of phosphatidylserine on the cell surface. Cell Death Differ..

[3] Suzuki J, Umeda M, Sims PJ, Nagata S (2010). Calcium-dependent phospholipid scrambling by TMEM16F. Nature.

[4] Yu K (2015). Identification of a lipid scrambling domain in ANO6/TMEM16F. Elife.

[5] Schreiber R (2010). Expression and function of epithelial anoctamins. J Biol Chem..

[6] Yang H (2012). TMEM16F forms a Ca2+ -activated cation channel required for lipid scrambling in platelets during blood coagulation. Cell.

[7] Marino G, Kroemer G (2013). Mechanisms of apoptotic phosphatidylserine exposure. Cell research.

[8] Watanabe R, Sakuragi T, Noji H, Nagata S (2018). Single-molecule analysis of phospholipid scrambling by TMEM16F. Proc Natl Acad Sci USA.

[9] Malvezzi M (2013). Ca2+-dependent phospholipid scrambling by a reconstituted TMEM16 ion channel. Nat Commun..

[10] Brunner JD, Lim NK, Schenck S, Duerst A, Dutzler R (2014). X-ray structure of a calcium-activated TMEM16 lipid scramblase. Nature.

[11] Gyobu S, Ishihara K, Suzuki J, Segawa K, Nagata S (2017). Characterization of the scrambling domain of the TMEM16 family. Proc Natl Acad Sci USA.

[12] Hu Y (2016). Scramblase TMEM16F terminates T cell receptor signaling to restrict T cell exhaustion. J Exp Med..

[13] Pardoll DM (2012). The blockade of immune checkpoints in cancer immunotherapy. Nat. Rev. Cancer.

[14] Headland SE (2015). Neutrophil-derived microvesicles enter cartilage and protect the joint in inflammatory arthritis. Sci Transl Med..

[15] Ousingsawat J (2015). Anoctamin-6 controls bone mineralization by activating the calcium transporter NCX1. Journal of Biological Chemistry.

[16] Anderson HC (2003). Matrix vesicles and calcification. Curr Rheumatol Rep..

[17] Ousingsawat J (2015). Anoctamin 6 mediates effects essential for innate immunity downstream of P2X7 receptors in macrophages. Nat Comms..

[18] Batti L (2016). TMEM16F Regulates Spinal Microglial Function in Neuropathic Pain States. Cell Rep..

[19] Imboden JB, Weiss A, Stobo JD (1985). The antigen recptor on a human T cell line initiates activation by increasing cytoplasmic free calcium. J Immunol..

[20] Yaradanakul A (2008). Massive Ca-induced membrane fusion and phospholipid changes triggered by reverse Na/Ca exchange in BHK fibroblasts. The Journal of general physiology.

[21] Lariccia V (2011). Massive calcium-activated endocytosis without involvement of classical endocytic proteins. The Journal of general physiology.

[22] Jiang, T., Yu, K., Hartzell, H. C. & Tajkhorshid, E. Lipids and ions traverse the membrane by the same physical pathway in the nhTMEM16 scramblase. *Elife* **6**, 10.7554/eLife.28671 (2017). 10.7554/eLife.28671 PMC5628016 28917060

[23] Williams K (1997). Interactions of polyamines with ion channels. Biochem J..

[24] Ye W (2018). Phosphatidylinositol-(4, 5)-bisphosphate regulates calcium gating of small-conductance cation channel TMEM16F. Proc Natl Acad Sci USA.

[25] Mirnikjoo B, Balasubramanian K, Schroit AJ (2009). Suicidal Membrane Repair Regulates Phosphatidylserine Externalization during Apoptosis. The Journal of biological chemistry.

[26] Lee SH, Meng XW, Flatten KS, Loegering DA, Kaufmann SH (2013). Phosphatidylserine exposure during apoptosis reflects bidirectional trafficking between plasma membrane and cytoplasm. Cell Death Differ..

[27] Gagnon E (2002). Endoplasmic reticulum-mediated phagocytosis is a mechanism of entry into macrophages. Cell.

[28] Cocucci E, Racchetti G, Podini P, Meldolesi J (2007). Enlargeosome traffic: exocytosis triggered by various signals is followed by endocytosis, membrane shedding or both. Traffic.

[29] Hilgemann DW, Fine M (2011). Mechanistic analysis of massive endocytosis in relation to functionally defined surface membrane domains. The Journal of general physiology.

[30] Bevers EM (1992). Defective Ca(2+)-induced microvesiculation and deficient expression of procoagulant activity in erythrocytes from a patient with a bleeding disorder: a study of the red blood cells of Scott syndrome. Blood.

[31] Bevers EM, Williamson PL (2016). Getting to the Outer Leaflet: Physiology of Phosphatidylserine Exposure at the Plasma Membrane. Physiological reviews.

[32] Ehlen HWA (2013). Inactivation of anoctamin-6/Tmem16f, a regulator of phosphatidylserine scrambling in osteoblasts, leads to decreased mineral deposition in skeletal tissues. J. Bone Miner. Res..

[33] Kumari S, Curado S, Mayya V, Dustin ML (2014). T cell antigen receptor activation and actin cytoskeleton remodeling. Biochim Biophys Acta.

[34] Wilson, E. B. *The Cell in Development and Heredity*. (New York: Macmillan, 1924).

[35] Cooper ST, McNeil PL (2015). Membrane Repair: Mechanisms and Pathophysiology. Physiological reviews.

[36] Idone V (2008). Repair of injured plasma membrane by rapid Ca2+ -dependent endocytosis. J Cell Biol..

[37] Cocucci E, Racchetti G, Meldolesi J (2009). Shedding microvesicles: artefacts no more. Trends Cell Biol..

[38] Jimenez AJ (2014). ESCRT machinery is required for plasma membrane repair. Science.

[39] Griffin DA (2016). Defective membrane fusion and repair in Anoctamin5-deficient muscular dystrophy. Hum Mol Genet..

[40] Wang TM, Hilgemann DW (2008). Ca-dependent nonsecretory vesicle fusion in a secretory cell. The Journal of general physiology.

[41] Sanjana NE, Shalem O, Zhang F (2014). Improved vectors and genome-wide libraries for CRISPR screening. Nature methods.

[42] Zufferey R, Nagy D, Mandel RJ, Naldini L, Trono D (1997). Multiply attenuated lentiviral vector achieves efficient gene delivery *in vivo*. Nature biotechnology.

[43] Trudgian DC, Mirzaei H (2012). Cloud CPFP: a shotgun proteomics data analysis pipeline using cloud and high performance computing. Journal of proteome research.

[44] Trudgian DC (2010). CPFP: a central proteomics facilities pipeline. Bioinformatics (Oxford, England).

[45] Craig R, Beavis RC (2004). TANDEM: matching proteins with tandem mass spectra. Bioinformatics (Oxford, England).

[46] Elias JE, Gygi SP (2007). Target-decoy search strategy for increased confidence in large-scale protein identifications by mass spectrometry. Nature methods.

[47] Trudgian David C., Ridlova Gabriela, Fischer Roman, Mackeen Mukram M., Ternette Nicola, Acuto Oreste, Kessler Benedikt M., Thomas Benjamin (2011). Comparative evaluation of label-free SINQ normalized spectral index quantitation in the central proteomics facilities pipeline. PROTEOMICS.

[48] Ho SN, Hunt HD, Horton RM, Pullen JK, Pease LR (1989). Site-directed mutagenesis by overlap extension using the polymerase chain reaction. Gene.

