# Author Correction: TMEM16F activation by Ca^2+^ triggers plasma membrane expansion and directs PD-1 trafficking

**DOI:** 10.1038/s41598-019-43808-0

**Published:** 2019-05-17

**Authors:** Christopher Bricogne, Michael Fine, Pedro M. Pereira, Julia Sung, Maha Tijani, Youxue Wang, Ricardo Henriques, Mary K. Collins, Donald W. Hilgemann

**Affiliations:** 1 UCL Cancer Institute, University College London, Gower St, London, UK; 2 University of Texas Southwestern Medical Center, Department of Physiology, Dallas, Texas USA; 3 MRC Laboratory for Molecular Cell Biology, University College London, Gower St, London, UK; 4 National Institute for Biological Standards and Control, Blanche Lane, South Mimms, Herts, UK; 5 Present Address: Okinawa Institute of Science and Technology, Onna-son, Okinawa, Japan

Correction to: *Scientific Reports* 10.1038/s41598-018-37056-x, published online 24 January 2019

The original version of this Article contained a typographical error in the spelling of the author Donald W. Hilgemann, which was incorrectly given as Donald Hilgemann.

As a result, the Acknowledgements section,

“HLA-A2 cDNA was a gift from Dr. Martin Pule, UCL. lentiCRISPR v2 plasmid was a gift from Feng Zhang, MIT. We thank David Escors, Djamil Damry, Mehdi Barachian, Mei-Jung Lin and Christopher Tie for help with this project and Dr. Criss Hartzell (Emory) for WT mouse TMEM16F plasmid. C.B. was supported by The Rosetrees Trust (M155), MRC Doctoral Training Grant (1132770), and the UCL Bogue Fellowship. D.H. and M.F. were supported by National Institutes of Health, USA (HL119843, T32DK007257). M.F., R.H. and P.M.P. were supported by Medical Research Council (MR/K015826/1) and Biotechnology and Biological Sciences Research Council (BB/M022374/1).”

now reads:

“HLA-A2 cDNA was a gift from Dr. Martin Pule, UCL. lentiCRISPR v2 plasmid was a gift from Feng Zhang, MIT. We thank David Escors, Djamil Damry, Mehdi Barachian, Mei-Jung Lin and Christopher Tie for help with this project and Dr. Criss Hartzell (Emory) for WT mouse TMEM16F plasmid. C.B. was supported by The Rosetrees Trust (M155), MRC Doctoral Training Grant (1132770), and the UCL Bogue Fellowship. D.W.H. and M.F. were supported by National Institutes of Health, USA (HL119843, T32DK007257). M.F., R.H. and P.M.P. were supported by Medical Research Council (MR/K015826/1) and Biotechnology and Biological Sciences Research Council (BB/M022374/1).”

In addition, the Author Contributions section,

“C.B., M.F. and D.H. designed and performed experiments, and wrote the paper. M.K.C. designed experiments and wrote the paper. P.M.P., J.S., M.T. and Y.W. designed and performed experiments. R.H. designed experiments.”

now reads:

“C.B., M.F. and D.W.H. designed and performed experiments, and wrote the paper. M.K.C. designed experiments and wrote the paper. P.M.P., J.S., M.T. and Y.W. designed and performed experiments. R.H. designed experiments.”

These errors have now been corrected in the PDF and HTML versions of the Article, and in the accompanying Supplementary Data file.

