# Supplementary material for: TMEM16F activation by Ca2+ triggers plasma membrane expansion and directs PD-1 trafficking
Source: Sci Rep. 2019 Jan 24;9:619. doi: 10.1038/s41598-018-37056-x (PMC6345885; doi:10.1038/s41598-018-37056-x)
Supplement: Supplementary file 1 — Supplementary data [file 41598_2018_37056_MOESM1_ESM.pdf]

TMEM16F activation by Ca^{2+} triggers plasma membrane expansion and directs PD-1 trafficking

Christopher Bricogne[1]*, Michael Fine[2]*, Pedro M. Pereira[3], Julia Sung[4], Maha Tijani[4], Youxue Wang[2], Ricardo Henriques[3], Mary K Collins[1,4,5]† and Donald W. Hilgemann[2]†

Author Affiliation:

[1] UCL Cancer Institute, University College London, Gower St, London, UK

[2] University of Texas Southwestern Medical Center, Department of Physiology, Dallas, Texas, USA

[3] MRC Laboratory for Molecular Cell Biology, University College London, Gower St, London, UK

[4] National Institute for Biological Standards and Control, Blanche Lane, South Mimms, Herts, UK

[5] Present Address since September 2016, Okinawa Institute of Science and Technology, Onna-son, Okinawa, Japan

Co-corresponding Author: mary.collins@oist.jp; Orcid ID 0000-0002-9539-0130.

Co-corresponding Author: donald.hilgemann@utsouthwestern.edu; Orchid ID 0000-0002-5288-5133

* These authors contributed equally to the work

† These authors contributed equally to the work

[Video file submitted separately]

Video S1. Ionomycin-induced membrane expansion and vesicle shedding in WT Jurkat T cells. WT Jurkat cells in Ringer's solution were stained with FM 4-64. 5μM ionomycin was added at 30s. The scale bar represents 10μm.

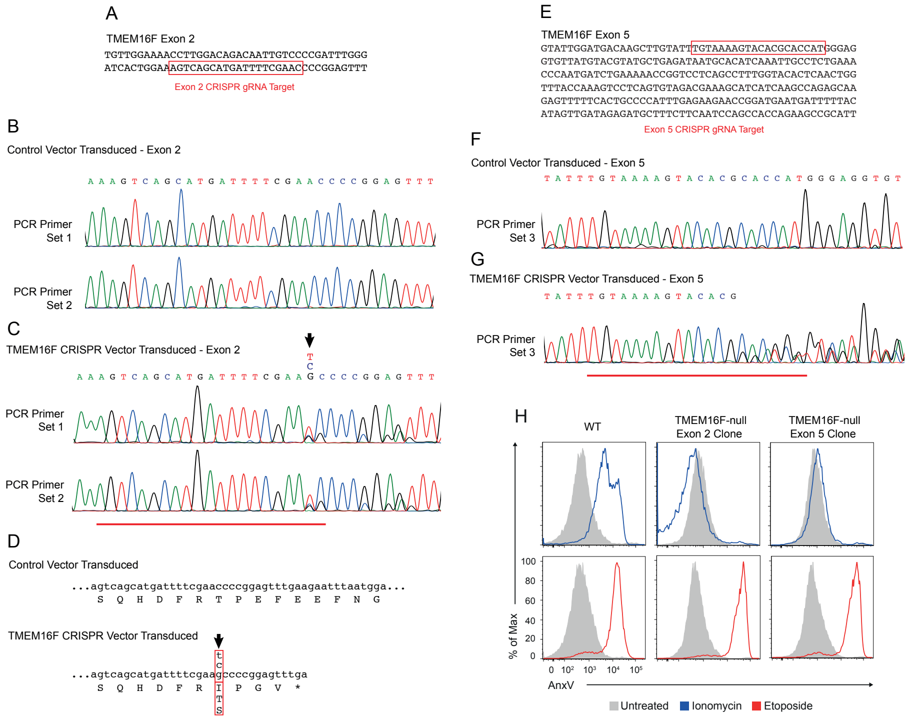

Figure S2. Generation of Jurkat T cells lacking TMEM16F.

The TMEM16F gene was edited using a lentiviral vector-based CRISPR-Cas9 system [40], targeted to TMEM16F exon 2 or exon 5. Exons were then amplified from genomic DNA, using primers described in Materials and Methods, and sequenced. **(A)** TMEM16F Exon 2 and target sequence is shown. **(B)** Two sets of primers were used to amplify TMEM16F Exon 2 from vector-only transduced Jurkat T cell genomic DNA. The two amplicons were then sequenced. **(C)** The same amplicons were sequenced from a clone transduced with TMEM16F Exon 2-targeted CRISPR-Cas9 lentiviral vector. **(D)** The result of the single nucleotide insertion on the amino acid sequence. A stop codon results 10 nucleotides downstream of the insertion that truncates TMEM16F from 910 to 50 amino acids. **(E)** TMEM16F Exon 5 and target sequence is shown. **(F)** Primers were used to amplify TMEM16F Exon 5 from vector-only transduced Jurkat T cell genomic DNA. The amplicons was sequenced. **(G)** The same amplicon was then sequenced from a clone transduced with TMEM16F Exon 5-targeted CRISPR-Cas9 lentiviral vector. Multiple changes were found in the TMEM16F alleles downstream of the target sequence. **(H)** WT, and TMEM16F-null exon 2 and exon 5 targeted cells were treated with 5μM ionomycin for 15 min or 2μM Etoposide overnight and stained with Annexin V and compared to untreated cells. WT cells treated with ionomycin show a clear increase in Annexin V binding to exposed phosphatidylserine. TMEM16F-null cells fail to show Annexin V binding during ionomycin treatment. Loss of TMEM16F does not interfere with

apoptosis-induced scrambling and PS exposure as overnight treatment of Etoposide shows similar increases in Annexin V positive cells for WT and both Exon2 and exon 5 targeted cells.

A

	WT		TMEM16F-null	
	peptides (unique)	% coverage	peptides (unique)	% coverage
**TFR1**	358 (43)	56	335 (43)	56
**Tetraspanin-7**	48 (2)	8.8	31 (2)	8.8
**CD1c**	8 (4)	8.4	7 (4)	11
**TMEM16F**	13 (6)	10	0	0

B

```
MKKMSRNVLL QMEEEEDDDD GDIVLENLGQ TIVPDLGSLE SQHDFRTPEF EEFNGKPDSL FFNDGQRRID FVLVYEDESR 80
KETNKKGTNE KQRRKRQAYE SNLICHGLQL EATRSVLDDK LVFVKVHAPW EVLCTYAEIM HIKLPLKPND LKNRSSAFGT 160
LNWFTKVLSV DESIIKPEQE FFTAPFEKNR MNDFYIVDRD AFFNPATRSR IVYFILSRVK YQVINNVSKF GINRLVNSGI 240
YKAAFPLHDC KFRRQSEDPS CPNERYLLYR EWAHPRSIYK KQPLDLIRKY YGEKIGIYFA WLGYYTQMLL LAAVVGVACF 320
LYGYLNQDNC TWSKEVCHPD IGGKIIMCPQ CDRLCPFWKL NITCESSKKL CIFDSFGTLV FAVFMGVWVT LFLEFWKRRQ 400
AELEYEWDTV ELQQEEQARP EYEARCTHVV INEITQEEER IPFTAWGKCI RITLCASAVF FWILLIIASV IGIIVYRLSV 480
FIVFSAKLPK NINGTDPIQK YLTPQTATSI TASIISFIII MILNTIYEKV AIMITNFELP RTQTDYENSL TMKMFLFQFV 560
NYYSSCFYIA FFKGKFVGYP GDPVYWLGKY RNEECDPGGC LLELTTQLTI IMGGKAIWNN IQEVLLPWIM NLIGRFHRVS 640
GSEKITPRWE QDYHLQPMGK LGLFYEYLEM IIQFGFVTLF VASFPLAPLL ALVNNILEIR VDAWKLTTQF RRLVPEKAQD 720
IGAWQPIMQG IAILAVVTNA MIIAFTSDMI PRLVYYWSFS VPPYGDHTSY TMEGYINNTL SIFKVADFKN KSKGNPYSDL 800
GNHTTCRYRD FRYPPGHPQE YKHNIYYWHV IAAKLAFIIV MEHVIYSVKF FISYAIPDVS KRTKSKIQRE KYLTQKLLHE 880
NHLKDMTKNM GVIAERMIEA VDNNLRPKSE
```

Figure S3. TMEM16F gene edited cells lack TMEM16F protein.

(**A**) Proteomic analysis of membrane proteins from Jurkat WT and exon 2-edited TMEM16F-null cells. Membranes were purified from the two cell lines, then proteins were extracted, digested with trypsin and subjected to mass spectrometric analysis. The number of peptides detected from four selected membrane proteins is shown, as is the percent of amino acids from each protein detected in the peptides.

(**B**) Distribution of unique TMEM16F peptides (shown in red) throughout the protein sequence.

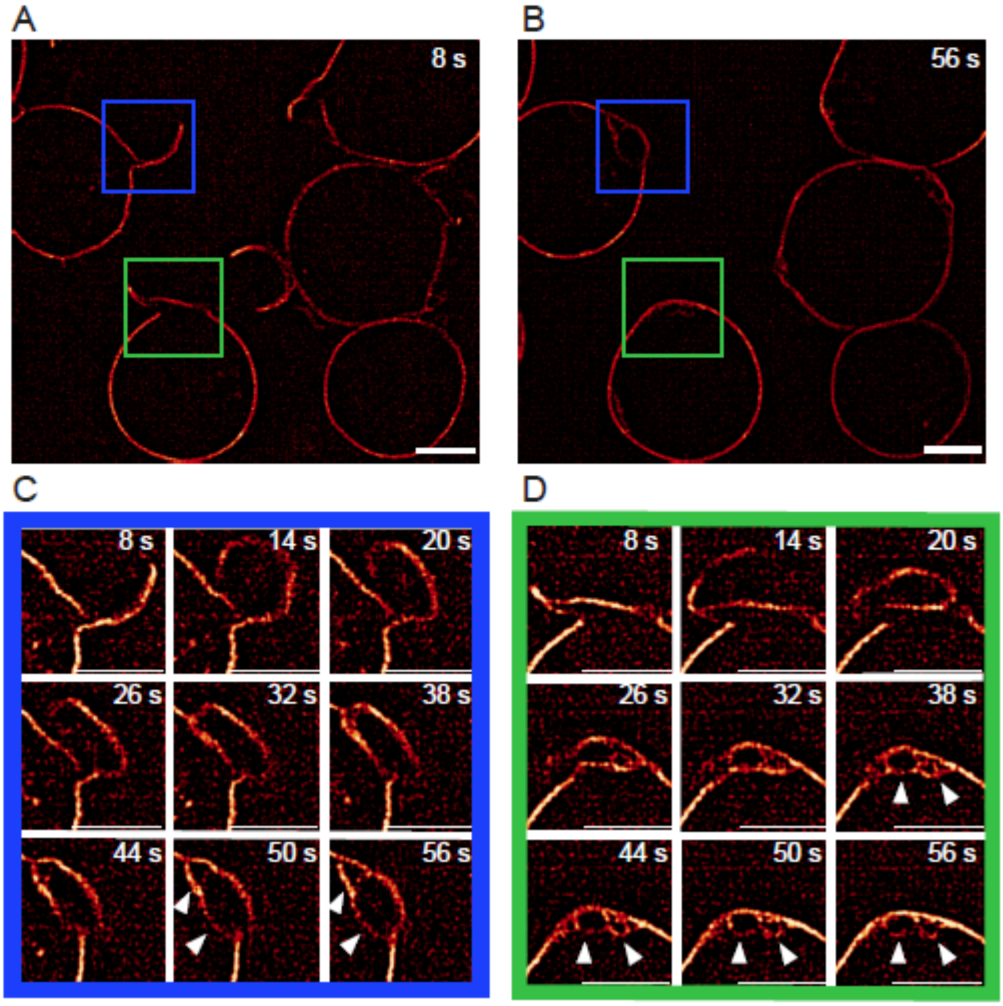

Figure S4. Intracellular vesicles derived from the plasma membrane after ionomycin treatment of TMEM16F-null Jurkat T cells

TMEM16F-null Jurkat T cells in Ringer's solution were stained with FM4-64 dye then treated with 5μM ionomycin for the period shown. SR-SIM microscopy images of FM4-64 dye distribution **(A)** and **(B). (C)** and **(D)** show the time course of MEND in two regions of interest (blue (C) and green (D)). Intracellular vesicles stained with FM4-64 are visible following MEND. The scale bars represent 10 μm.

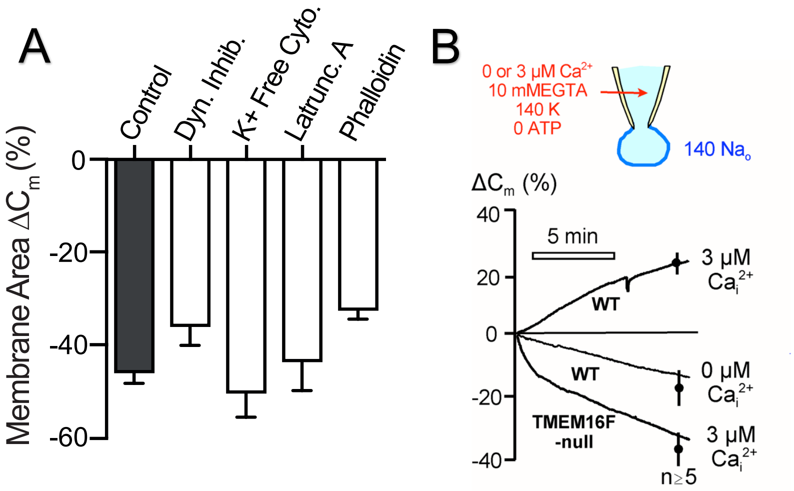

Figure S5. No effect of endocytosis inhibitors on plasma membrane decrease induced by ionomycin in TMEM16F-null Jurkat T cells, and membrane trafficking induced by physiological calcium levels.

A.Percentage change in C_m 30s after ionomycin treatment of TMEM16F-null Jurkat T cells. Results are from left to right: KCl/NaCl solutions (Control), control solutions with 3μM cytoplasmic dynamin inhibitory peptide, symmetrical K+-free NmAs solutions, control solutions with 3μM Latrunculin A, and control solutions with 10μM cytoplasmic Phalloidin. (C, D represent mean values with SEM, n=7) **B** Capacitance recordings in Jurkat cells employing a cytoplasmic solution modified to contain 10 mM EGTA with either no added $CaCl_2$ or 8 mM $CaCl_2$ to generate a free Ca^{2+} of 3 μM at pH 7.0. 35°C. In WT Jurkat cells with 3 μM free Ca^{2+}, membrane area increases on average by 25% over 10 min after cell opening, while in the absence of cytoplasmic Ca^{2+} membrane area decreases by 18%. In TMEM16F-null cells with 3 μM free Ca^{2+}, membrane area decreases on average by 37% over 10 min. In each case, n >=5.

[Video file submitted separately]

Video S6. Ionomycin-induced PS exposure detected with K7r in WT Jurkat T cells. WT Jurkat cells in Ringer's solution were stained with 3µM K7r. 5 µM ionomycin was added at 50s. The scale bar represents 10µm.

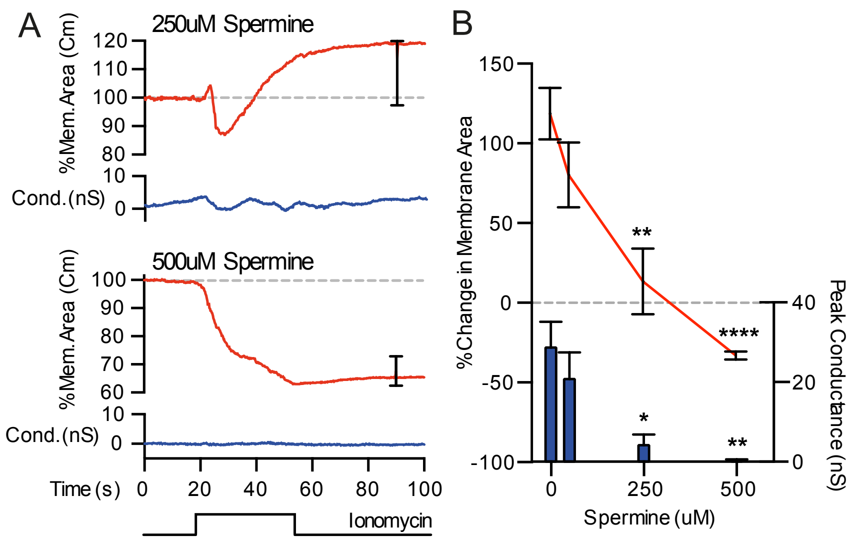

Figure S7.

(A) Spermine at 250 and 500µM was added to cytoplasmic solutions. Cells were held for at least 300s before ionomycin treatment. Single experimental traces are given with mean C_m change and SEM. n>7. **(B)** Change in membrane area (ΔC_m in %) and peak G_m magnitudes were determined during ionomycin application. C_m and G_m responses were compared to control responses without spermine (* $p \leq 0.05$, ** $p \leq 0.01$, ****$p \leq 0.0001$).

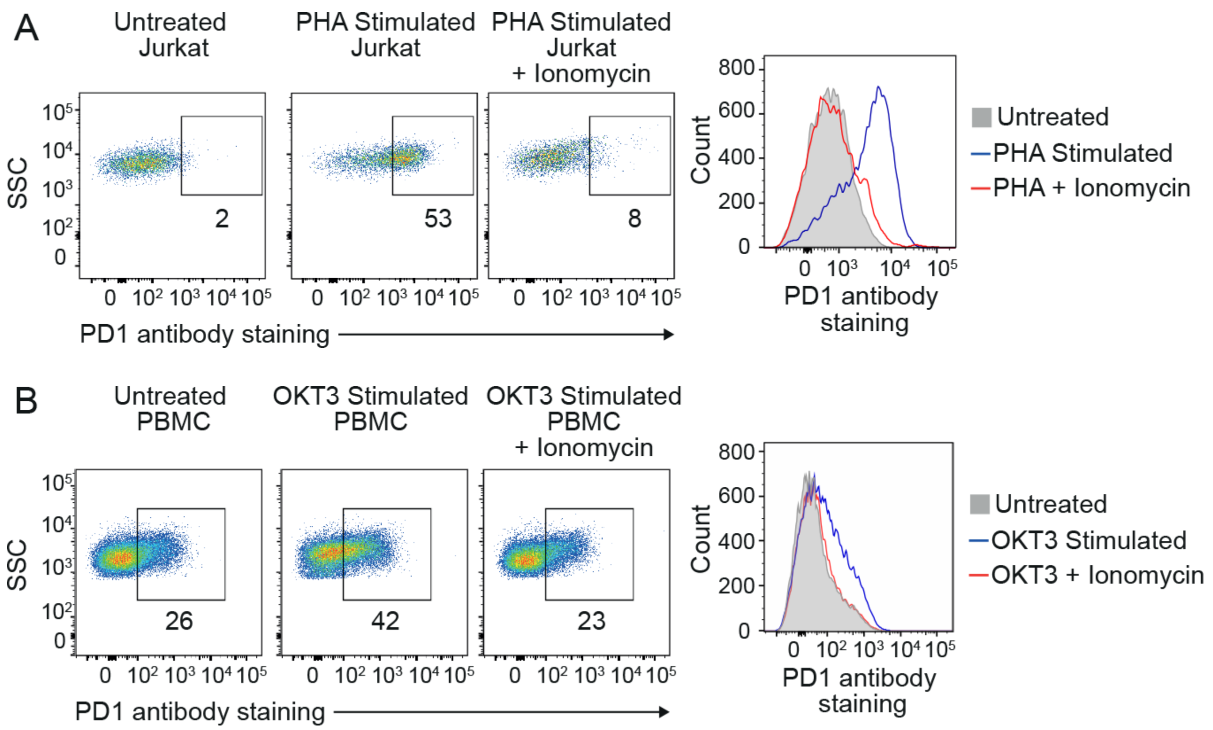

Figure S8.

(A) Jurkat T cells were treated for 24h with phytohaemagglutinin (PHA, Sigma) at 5µg/ml. Some cells were then incubated with 5 µM ionomycin for 15 minutes, after which PD-1 surface expression was measured by flow cytometry. A representative experiment from 3 replicates is shown. **(B)** Peripheral blood mononuclear cells (PBMC) from a healthy donor were treated for 24h with 5µg/ml of an anti-CD3 antibody (OKT3, eBioscience). Some cells were then incubated with 5 µM ionomycin for 15 minutes, after which PD-1 surface expression was measured by flow cytometry. A representative experiment from 3 replicates is shown.

[Video file submitted separately]

Video S9. In WT Jurkat T cells PD-1 is shed following ionomycin treatment. PD1- mCherry expressing WT Jurkat T cells were imaged by SR-SIM Microscopy. Ionomycin was added to the edge of the coverslip 10s before the video starts. Scale bar represents 10 µm.

[Video file submitted separately]

Video S10. In TMEM16F-null Jurkat T cells PD-1 is endocytosed following ionomycin treatment. PD1-mCherry expressing TMEM16F-null Jurkat T cells were imaged by SR-SIM Microscopy. Ionomycin was added to the edge of the coverslip 10s before the video starts. Scale bar represents 10μm.
